# T92. QUITLINK: ACCESSIBLE SMOKING CESSATION SUPPORT FOR PEOPLE LIVING WITH SEVERE AND ENDURING MENTAL ILLNESS

**DOI:** 10.1093/schbul/sby016.368

**Published:** 2018-04-01

**Authors:** Amanda Baker, Ron Borland, Billie Bonevski, David J Castle, Jill Williams, Catherine Segan, Peter Kelly, Alyna Turner, Lisa Brophy, Rohan Sweeney

**Affiliations:** 1 University of Newcastle; 2 Cancer Council Victoria; 3 St. Vincent’s Hospital, University of Melbourne; 4 UMDNJ-Robert Wood Johnson Medical School; 5 University of Wollongong; 6 Deakin University; 7 University of Melbourne

## Abstract

**Background:**

People with severe mental illness (SMI) typically die 20 years earlier than the general population, largely due to smoking related diseases. Their smoking rate is alarmingly high and persistent, which contrasts sharply with the steady decline in the general population’s smoking rate. Smokers with SMI are equally motivated to quit smoking, but report less encouragement to quit by health professionals and are less able to succeed. When engaged in a program, some can quit successfully, but at lower rates than for the general population. Evidence-based smoking cessation interventions, such as quitlines, are underutilised by smokers with SMI. There is an urgent need to develop highly accessible, appropriately tailored cessation services for smokers with SMI to which mental health services can routinely refer smokers, and to explore why low smoking cessation rates persist among people with SMI receiving cessation treatment.

Quitlink will utilise peer workers to identify, support, and refer smokers with SMI in mental health services to Quitline, who will deliver a tailored, proactive and accessible smoking cessation intervention. Additionally, we wish to investigate participant and health worker perceptions of the support provided by Quitlink, the nature of barriers encountered and their impact on initiating and succeeding with cessation.

**Methods:**

Design: A multi-centre prospective, randomised, open, blinded endpoint (PROBE) design will be utilised to compare standard smoking care alone against Quitlink. Assessments will be conducted at baseline, end of treatment, 3 months post treatment and 6 months post treatment.

Setting and sample: 382 smokers will be recruited from three mental health services in Victoria: Mind Australia, Neami and Melbourne’s St Vincent’s hospital.

**Intervention:**

The manual guided Quitlink intervention consists of standard smoking care plus: Referral to Quitline, Quitline counselling, Quitline provision of NRT, Quitline engagement with mental health services, Quitline engagement of support network and peer worker help with participant contact.

**Results:**

As recommended by the Society for Research on Nicotine and Tobacco expert workgroup, the primary outcome measure in this study will be 6 months prolonged abstinence at the 8-month follow-up (allowing participants up to 2 months to stop). Prolonged 6-month abstinence will be defined as per the Russell Standard, i.e. self-report of abstinence for 6 months (allowing up to 5 cigarettes in total) and biochemical verification of self-reported abstinence (validated in a face-to-face visit using expired CO monitoring using a Micro+ Smokelyzer, with a reading of over 8ppm defined as a treatment failure).

**Discussion:**

Outcomes of the project will determine effectiveness and cost-effectiveness of a novel and highly translatable intervention resulting from linking two existing services (Quitline and mental health peer workers). This will be the world first RCT of a Quitline intervention delivered to people with SMI that also includes a concurrent economic evaluation. As smoking is the leading cause of preventable death in people with SMI, if Quitlink is shown to be effective, it has the potential to greatly improve individuals’ longevity, quality of life, mental health and reduce health care costs. This is an innovative and practical service delivery model that has the potential to ensure that smokers with SMI have access to best-practice smoking cessation treatment. Secondly, regardless of effectiveness outcomes, the project’s qualitative study will provide greater insights into the barriers faced by smokers with SMI and will assist in the development of even more effective interventions.

